# Reforming Italy’s long-term care system: the role of barriers to and drivers of the use of services at the local level

**DOI:** 10.3389/fpubh.2025.1575330

**Published:** 2025-04-28

**Authors:** Sara Santini, Gianluca Busilacchi, Giovanni Lamura, Federico Sofritti, Antonio Pacifico, Ramona Bongelli, Carmela Guarascio

**Affiliations:** ^1^Centre for Socio-Economic Research on Aging, IRCCS INRCA-National Institute of Health and Science on Aging, Ancona, Italy; ^2^Department of Economics and Law, University of Macerata, Macerata, Italy

**Keywords:** long-term care system, barriers to long-term care services, drivers to long-term care services, long-term care reform, care dyad, older people with long-term care needs

## Abstract

**Background:**

In Italy, population ageing is causing an unprecedented demand for long-term care (LTC) services, that led to the recent national reform of the LTC system (Law n. 33/2023). Since LTC services are provided by regional authorities, identifying drivers of and barriers to their use by older people and their family caregivers locally is very important to identify the mismatch between national regulation and local demand of these services.

**Methods:**

To this purpose, in 2019-2020, 450 family caregiver (FC)-older care recipient (OCR) dyads from 13 healthcare districts of the Marche region (Central Italy) were surveyed. A Two-step Bayesian Multiclass procedure was used for the analysis. The main drivers of the use of healthcare services are FC’s age and gender (being a man), and OCR’s age and level of disability.

**Results:**

The main barrier to the use of private services is their cost, while for the public ones is their unavailability. The most common private service is represented by migrant care workers (MCWs), hired privately by the older people’s families.

**Conclusion:**

Findings suggest that the recent national LTC reform in Italy does not seem to have fully captured the LTC needs of older people, and some policy suggestions are therefore provided in this regard.

## 1 Introduction

This study is aimed at investigating the barriers to and drivers of the use of health and long-term care services (in the following “services,” when not differently specified) by older adults with long-term care needs and their family caregivers living in Marche, a region in Central Italy. Healthcare services include outpatient visits, day hospitals, emergency department access, and general practitioners, i.e., services ensured by Italy’s regional health systems to all citizens and not only to those with chronic disease. On the contrary, LTC services specifically target people with disability and long-term (chronic) diseases such as Parkinson’s, dementia, and any type of physical impairment requiring medical, nursing, household care, and personal assistance over a long period of time. These include residential care facilities, home care, monetary transfers, and the private work of family care assistants.

A peculiarity of Italy’s Healthcare System is that it is actually made of 21 healthcare systems, i.e., as many as the 19 regions and two autonomous provinces making up the country, given the autonomy that these authorities have in managing the provision of healthcare and LTC. On this background, we chose the Marche region as a case study for two main reasons. The first is that it has one of the highest shares of older population in the country, with 26% of its citizens being aged 65 years and over (1, 2), i.e., two percentage points above the national average (24%), and among the highest in Europe (whose average is 21.3%) (3). The second reason lies in the middle position of the Marche region in the ranking of the healthcare performance among Italy’s regional health systems (4). Given this position, the Marche region can constitute a benchmark for both the most (the North-Eastern) and the least virtuous regions (the Southern ones) in the ranking.

In the following sub-paragraphs, we will highlight the main characteristics of the LTC demand and supply in Italy, describe how the recent LTC reform tries to improve care provision, and identify barriers and drivers to the use of LTC at the macro, meso, and micro societal level.

### 1.1 The Italian healthcare system and the LTC reform

In Italy, the share of people aged 65 years and over increased by more than 10% between 2002 and 2021, raising from 12.4 to 23.5%, with projections reaching 34.9% by 2050 (5). In 2019, 32.4% of older Italian population lived with severe chronic and/or multiple diseases. More than half of them had at least three chronic diseases, reducing their autonomy in performing the activities of daily living (ADL) to the point of requiring assistance from others (6). All this entails also an unprecedented increase in the request for support for carrying out the instrumental activities of daily living (IADL) and for meeting older people’s physical and psychological needs (7).

Despite this increase in the demand side, LTC still remains a residual aspect of Italy’s welfare system, and it is funded and implemented by different national, regional, and local institutions (53). While social services are delivered by municipalities, healthcare and LTC services are delivered by local healthcare authorities (“*Aziende Sanitarie Locali*”), according to the policy directions adopted at the regional level. Indeed, Italian regions, despite acting according to a legislative national framework including the LTC reform described further below, can independently decide how to use the economic resources allocated to them by the State to address the local population’s care demand (8), thus constituting de facto 21 different regional systems governed by politicians and very variegated in terms of territorial size and population characteristics. This autonomy often translates into substantial inequalities in accessing care services across regions and results in a fragmentation of funding and responsibilities that prevents a full and proper integration of social and health services in terms of implementation (9).

Another key characteristic of Italy’s welfare system is its “familistic” approach (10) that entrusts family caregivers with the responsibility of caring for frail, mainly older people with LTC needs, and supporting them primarily via monetary transfers rather than in-kind services (11). The main cash benefit in Italy is the State’s “Indennità di accompagnamento” (IDA) (“care allowance”), a non-means-tested monetary benefit (financed through general taxation) granted to people affected by a severe disability. In 2019, it absorbed 52% of the resources allocated to LTC, compared to a European average of around 26% (12). The presence of other similar cash benefits provided at the regional level, mostly means-tested, confirms the importance of monetary benefits in the Italian LTC system (13).

Conversely, public resources invested in home care (“*Assistenza Domiciliare Integrata*”: ADI) are limited: in 2019, they accounted for 19.5% of total public spending in this area, against a European average of 25.5% (12). Currently, home care provided by regional health units lasts for a maximum of 3 months, while LTC needs affect older people on average for several years. Regions also provide home-based nursing services (e.g., medication, catheter changes, and similar) but without addressing LTC-related needs such as information, counseling, and psychological support for family caregivers.

As for residential care, it is still underdeveloped in Italy and is again characterized by a strong regional variability. In 2021 (most recent available data), compared with a national average of 70 beds per 10,000 residents, in the North-East, the availability reached 100 beds, i.e., three times higher than in the South (33.4), while an intermediate situation emerges for the Islands (Sardinia and Sicily), with 51 beds, and the center (including the Marche region), with 56 beds. The North-West is more similar to the North-East, with 97 beds per 10,000 residents (1, 2) (p. 290).

Regarding nursing homes (“*Residenza Sanitaria Assistenziale*”: RSA), i.e., residential facilities offering a higher level of medical care in addition to accommodation and meals, the monthly fee for beneficiaries reaches on average EUR 2,000, with strong differences across regions. This amount includes (Law 502/92 on “Reorganization of healthcare regulations”) a medical component (generally 50% of the whole), borne by the regional health system, and a means-tested social/accommodation component (the other 50%), borne by the beneficiary. When the patient is entitled to the State Care Allowance (Indennità di Accompagnamento: IDA), this can be used to pay the monthly expenditure for the nursing home.

The different expenditures of regional authorities for residential care are not congruent with the distribution of chronicity, nor with older people’s health condition or income, but rather explained primarily by the regions’ overall wealth and financial resources (i.e., higher in wealthier regions, such as Trentino Alto-Adige) and by the rate of unemployed women as potential informal (family) caregivers (14). This causes remarkable inequalities among older citizens living in different regions for the per-capita expenditure in residential care.

The scarcity of in-kind LTC services (e.g., ADI and residential care) and the availability of a non-restricted (nor monitored) monetary transfer, such as the IDA, push many families to hire private non-professional care assistants to guarantee home-based, tailored, and around-the-clock assistance and supervision to older relatives with LTC needs (15, 16). These private care assistants, often referred to as “migrant care workers” (MCWs) because most of them have a migration background, help older people in performing ADL and IADL, allowing family caregivers to reconcile care and work, thus keeping alive Italy’s traditional picture of family care for aging relatives. All this at a rather relatively low cost, in unfair and often under partially or totally undeclared work conditions (17, 18), and indirectly encouraged by generous and unconditional cash benefits like the IDA (19, 20).

Despite this background, Italian families who decide to hire a family care assistant still have to bear certain costs themselves. They include the salary, which varies according to the care worker’s level of training, the weekly number of working hours, and whether s/he lives with the older person being cared for. For example, an untrained, live-in care worker receives a salary of approximately EUR 1,127 per month. To this salary, the 13th salary, holidays, end of service payment (“*Trattamento di fine rapporto*”), and the board and lodging allowance (if any) have to be added, which for 2024 was €196, for a total monthly expenditure of approximately EUR 1,450.

All the policy measures reported above targeted people with disability. Noteworthy, in Italy, there is no national law recognizing family caregivers’ role and promoting their rights, but only regional laws in 10 regions (including Emilia-Romagna, which approved the first regional bill in this regard in 2014). This means that there is no integrated national system of support explicitly addressing family caregivers, offering them, for instance, training, counseling, psychological support, and/or respite care.

As for working caregivers, according to Law 104/1992, care leave is granted only to public and private employees (i.e., excluding the self-employed and those employed in domestic and household services) caring for relatives with a severe disability. Working caregivers are entitled to two different types of care leaves: 3 working days of paid short-term leave per month, under the condition that the FC is a close relative of the person with the disability (even when not co-habiting); and up to 2 years of paid long-term leave (only in case of cohabitation). The latter leave is paid 100% of earnings up to an annual ceiling (adapted over time according to inflation).

To counteract the limitations of the Italian healthcare system(s) described above, after approximately 20 years of policy inertia (21), on 31st March 2023, Law n. 33 “Deleghe al Governo in materia di politiche in favore delle persone anziane” (Delegations to the Government on policies in favor of older persons) came into force. This law, whose first implementing decree was approved in March 2024, is aimed at reforming the Italian healthcare policies for frail older people and overcoming their weaknesses, i.e., services fragmentation and scarcity, thus improving the response to the LTC needs of older people via more accessible, high-quality, and timely services (22).

The key components of the LTC reform stand on four main pillars. The first one is a new model of governance of the LTC services, looking at LTC needs in later life in a more comprehensive and specific way. The body to achieve this goal was the “National System for the Older Population with LTC needs” (“*Sistema nazionale per la popolazione anziana non autosufficiente*”: SNAA), entitled to plan and govern holistically all health, social, and welfare measures adopted by the State, regions, and municipalities, including the economic benefits provided by Istituto Italiano di Previdenza Sociale (INPS) (i.e., the National Security Authority), such as the IDA. The second pillar is a new home care model for aging in place, which should have foreseen the strengthening of public home healthcare (*Assistenza Domiciliare Integrata*: ADI) and the integration of social and health support measures. The third pillar of the law is a new residential care model that should have included social and health activities. The fourth pillar consists of the reform of the IDA, to be graduated according to the intensity of a person’s care needs and granted as a choice between monetary transfer and in-kind services. Moreover, the reform aims to achieve full integration of social and health services for non-self-sufficient older adults also through a system of multidimensional assessment of the individuals’ condition that sees the “Single Point of Access” (*Punto Unico di Accesso*: PUA) as the only gateway to the care system. Currently, only some of these components have started to be implemented, and many observers fear that the financial constraints currently affecting the Italian State may jeopardize the full implementation of the reform’s main goals (23).

### 1.2 Barriers and drivers to the use of LTC services

In recent years, several studies have analyzed the determinants of access to long-term care services across European countries and within Italy (12, 24, 25). However, the interplay between socioeconomic status, regional policy design, and household strategies remains underexplored, especially in fragmented welfare settings such as the Italian one. This review synthesizes key contributions on barriers and drivers to LTC service utilization at the macro, meso, and micro levels, with particular attention to their policy implications.

Adequate support is crucial to mitigate and postpone negative effects that can be raised by the lack of efficient and timely services for older people with LTC needs, e.g., faster physical decline, mental health issues, and higher mortality rates (26, 54). There are many intertwined social and psychological/cultural factors that influence the access to care services by older people with LTC needs and their family caregivers at the macro, meso, and micro levels.

At the macro level, some cross-national studies confirm the weight of welfare regimes and LTC systems in deploying inequalities and stress that more developed and all-embracing LTC systems characterized by a high number of care services can decrease the risk of inequalities in access to and use of the services (24, 27). In fact, the Northern European welfare state model shows a higher propensity toward care equity, whereas Southern (familistic) and Continental models suffer from a certain care inequity when it comes to responding to LTC needs of older people (24, 27).

At the meso level, one of the main barriers is the scarcity of in-kind and tailored public formal services that push households to purchase private and informal services (25, 28). Moreover, the lack of information on services-specific features and the complexity of the access procedure can discourage older people from benefitting from formal services (29). The share of households needing LTC that do not use public professional home care because they find them insufficient is significantly higher in Italy (30.3%) compared to the EU average of 9.7% (12).

At the micro level, the socioeconomic condition of individuals can also affect the utilization of public LTC services (54). In familistic care regimes, such as Italy, people with higher income tend to use more private formal services (e.g., private nursing homes), whereas private informal care (e.g., MCWs) is more widespread among people with lower income (55). Moreover, older people living alone may experience barriers to accessing community services because they do not have sufficient information and limited mobility (because of none to accompany them to the services) (30). In addition, the lack of support needs recognition by the family caregiver of older people with dementia (31), the reluctance of the latter, who often refuse services (32), and the stigma around dementia (33) can limit the use of LTC services. Finally, regardless of the kind of disease of the older people, the lack of trust between the older person and the family caregiver may be a barrier to accepting homecare services (34).

Looking at the Italian context, over the last 20 years, low educational level, low income, and living in the Southern regions have been underlined as the main obstacles to access public healthcare services (35, 36), including dental care (37). Recently, a long waiting list for specialist services was added to the list of barriers (38). The main reasons for reporting unmet needs for medical care in Italy are the waiting list and the high cost of the services (39). From 2019 to 2023, the percentage of Italian citizens who had to forego examinations or screenings due to financial problems, waiting lists, or access difficulties rose from 7 to 7.6%, reaching approximately 4.5 million people in 2023. In the Marche region, the percentage passed from 7 to 9.8% in the same time range (1, 2) (p. 288). Noteworthy, the rate of foregoing health services rises with the increasing age of citizens (1, 2).

Moreover, in Italy, structural and organizational limitations of the current offer of LTC services can be paired with cultural attitudes rooted in the familial care tradition and filial obligations (40), which may be an additional barrier to the use of LTC services, e.g., the stigma surrounding residential/institutional care often accompanying family caregivers’ sense of guilt (41). In fact, we are witnessing a progressive increase in the use of private healthcare services (42) and in long-term care insurance (43), contrary to the principle of equity on which the National Health System has been originally based.

As indicated above, many studies have analyzed the factors influencing access to and use of healthcare services at the national level, both in Italy and in Europe. However, this phenomenon is still underinvestigated at the regional level and not focused on LTC services, e.g., care facilities and home care. Especially in countries such as Italy, where LTC services are administered by regional/local authorities, it is instead crucial to study the regional context to better understand the source of disparities in the use and access to services. For these reasons, this study aims to fill this gap through the identification of drivers and barriers to the use of healthcare services by older adults with LTC needs in Italy, to provide policy suggestions to make LTC delivery and resource allocation more efficient at the local level.

## 2 Materials and methods

### 2.1 Study design

Starting from the hypothesis that macro, meso, and micro social factors may influence the use of public and private care services, the research question that informed our study is the following: What are barriers and drivers to the use of public and private LTC services by older people with LTC needs and their family caregivers living in the Marche region, at different societal level?

### 2.2 Sampling

The study is part of a survey conducted in the Autumn 2019/Winter 2020 that involved 450 caregiver-cared-for dyads from 13 healthcare districts of the Marche region, recruited following a convenience/purposive sampling procedure (44). In fact, possible participants were enlisted through regional pensioners’ associations and Trade Unions, with the survey administration conducted by trained personnel from these organizations. They were screened according to the inclusion criteria, i.e., being aged 75 years or older, receiving the IDA (*Indennità di accompagnamento*), or reporting a score below 9 on a 12-item scale gauging autonomy in Instrumental Activities of Daily Living (IADL).

Inclusion criteria for study participation encompassed individuals who willingly signed the informed consent, expressed their voluntary commitment to the study, and met specific conditions.

Upon meeting the criteria for eligibility, older people were requested to designate their primary family caregivers, who were mostly family members such as spouses or sons/daughters. When family members were unavailable, private (migrant) care workers, predominantly those providing live-in support, were incorporated as the second component of the caregiving dyad.

### 2.3 Ethics statement

Explicit written consent was sought from respondents, and all data were gathered anonymously, adhering to the guidelines outlined in EU Regulation No. 679 of the European Parliament and of the Council of 27 April 2016, as well as the Helsinki Declaration (2013). The study was submitted for approval at the Ethics Committee of the National Institute of Health and Science on Ageing (INRCA), which on 25th May 2020 by an official e-mail communication deemed approval unnecessary since the investigation did not imply the involvement of clinical patients.

### 2.4 Data collection tools and outcome measures

Data were collected through a common assessment tool made of two questionnaires, one targeted to OCRs and another one to FCs. The first questionnaire, in addition to questions collecting sociodemographic information, embedded questions on respondents’ perceived health condition (ad-hoc question) and level of autonomy (Barthel index).

The Barthel index score ranges from 0 to 99, with scores of 0–20 indicating total dependence and 91–99 slight dependence (45, 46). Thus, the higher the score, the greater the autonomy of the care recipient.

The second questionnaire explored FCs’ burden, informal support, and work–life balance. Both the questionnaires asked about the use of public and private LTC services through ad-hoc multiple-choice and open-ended questions.

In the analysis reported in this study, the use of LTC services represents the outcome variable. It was assessed through the question, “Which of the following services did you use in the last year?.” Respondents could choose from a list of private and public care services. The answers to this question were dichotomized into used/not used of private vs. public services. Respondents could also specify the frequency of use, selecting weekly, monthly, or less frequently, and the level of satisfaction with the service, expressed in a five-point Likert scale ranging from 1 = very unsatisfied to 5 = very satisfied.

Independent variables are detailed in Annex 1 (Supplementary materials). They were, e.g., older care recipient’s age, living condition (dichotomized into “living alone” vs. “with the family caregiver” vs., “with others,” including migrant care workers), and level of dependency (Barthel Index).

### 2.5 Analysis

In this study, a Two-step Bayesian Multiclass (TBM) procedure is used, combining a first-step Bayesian strategy for selecting the only (potential) predictors affecting the outcomes with a frequentist second-step procedure for estimating the parameters of a binary logistic regression.

The first step focuses on the Bayesian Information Criterion (BIC) for model selection among a finite set of models. It provides a way to balance model fit and model complexity, helping to guard against overfitting. This latter refers to the problem that more complex models (including a sufficiently large number of predictors) tend to fit better than simpler models (fewer predictors). Thus, the first step entails finding a pool of predictors with highly strong explanatory powers on the potential outcomes of interest (or dependent variable). The predictors that fit the data better will be included in the shrinking procedure; otherwise, they will be ruled out.

The second step addresses a bivariate logistic regression on the subset of covariates obtained in the first step. The statistical-econometric software used for the analysis is RStudio, an open-source integrated development environment for R and Python (two similar software). The latest version used is 2024.04.0.

The main motivation to adopt a TBM procedure lies in the complexity of the problem aimed to address. The study investigates the barriers and drivers influencing the use of long-term care (LTC) services, which involve multiple interacting factors with varying levels of importance. Traditional logistic regression models, while effective in simple classification tasks, can struggle in high-dimensional settings where multiple predictors contribute in different ways to the outcome. To overcome these challenges, a TBM approach is then addressed consisting of two sequential steps: (i) instead of arbitrarily including all possible covariates, a Bayesian Information Criterion (BIC)-based selection process is used to identify the most relevant predictors. This intermediate step can deal with overfitting, ensuring that only the strongest explanatory variables are retained while filtering out less relevant ones; (ii) once the key predictors are identified, logistic regression is applied to estimate the final model parameters. This final step ensures interpretability, as logistic regression coefficients remain intuitive and comparable across models. In contrast, a standard logistic regression model requires either including all potential predictors, which increases the risk of overfitting and multicollinearity, or manually selecting them, introducing subjectivity. Machine learning (ML) methods, while powerful, often lack transparency due to their complexity, making it difficult to clearly identify the individual contribution of each predictor to the final outcome. This characteristic, known as the “black-box” problem, limits their interpretability, which is a critical aspect when dealing with policy-oriented research. Additionally, machine learning methods often require large datasets to generalize well. Given that the study is based on 450 dyads, the Bayesian regularization in TBM is particularly beneficial, as it is designed to perform effectively with smaller samples.

## 3 Results

### 3.1 Sample description

Supplementary Table reports the sociodemographic characteristics of FCs and OCRs answering the questionnaire.

The FCs’ group also includes 11 live-in MCWs. We decided to consider them as family members because, de facto, they replace the natural OCRs’ sons and daughters living away. Moreover, since this study is focused on the use of the services and not on the relationship with the caring dyad or the care burden, including the small group of MCWs in the overall sample of FCs cannot affect the results at all.

The FCs’ sample comprises 71.43% women, the mean age is 66 years, and 96.76% is Italian. The respondents of other nationalities are 13 MCWs, except for one family caregiver from Albania and another one from Morocco caring for older parents; 69.25% of respondents are married, and 43.89% achieved a high school degree.

Concerning the relationship with the care recipient, 60.50% of respondents are sons and daughters. More than half of the sample lives with the OCR (56.97%). On average, the respondents are caring for their loved ones for more than 7 years (approximately 86 months), providing 62.06 h of care per week. Not surprisingly, given the intense care provided and the mean age, only 28.34% have paid work (working caregivers).

The OCRs’ mean age is 85.8, and they are mainly women (71.43%) and most Italian (99.55%). More than half are widowed (58.96%) and have a primary school education (61.90%), while 14.06% have no qualification.

Concerning the living conditions, 36.96% live with a daughter or son, 35.15% with a spouse or partner, 27.44% with the MCW, and only 14.97% live alone. The perceived health status is “fair (reasonable)” for 39.46 respondents and bad for approximately 21%. This perception is mirrored by the Barthel Index, according to which 44.14% of older care recipients have medium-high dependency levels (score 21–90) and 45.12% of OCRs hired an MCW.

### 3.2 Use of long-term care services by satisfaction

We analyzed the frequencies in the use of public and private healthcare services by the level of users’ satisfaction. The three most used services by OCRs are: (a) private home care, e.g., specialist practitioners, nurses, physiotherapists, and migrant family care assistants; (b) public home care, e.g., nurse, physiotherapist, and meal delivery (ADI); (c) public outpatient services, e.g., general practitioner, physiotherapist, social workers, and nurse (Table 1). The association between the frequency of use and the level of satisfaction is highly significant (*p* = 0.000).

**Table 1:** Logistic function: frequency of services use by satisfaction.

Predictors	Estimate	SE	*z*-value	Pr(>|z|)
Frequency in the use of private home care	3.943	0.782	5.041	0.000***
Private home care satisfaction	1.148	0.408	2.814	0.000***
Private home care dissatisfaction	1.512	0.486	3.115	0.000**
Frequency in the use of public home care (ADI)	3.346	0.581	5.755	0.000***
Public home care satisfaction	1.867	0.592	3.156	0.000***
Public home care dissatisfaction	−3.320	0.437	−7.596	0.000***
Frequency in the use of public outpatient services	2.011	0.316	6.362	0.000***
Public outpatient services satisfaction	−1.138	0.356	−3.198	0.000***
Public outpatient services dissatisfaction	−1.118	0.652	−1.713	0.044**

Here, “Coefficients” refers to the covariates; “Estimate” refers to the estimated best final predictors; “SE” stands for Standard Error; “z-value” denotes the test statistic obtained for each predictor (the ratio between “Estimate” and “SE”); and “Pr(>|z|)” refers to the associated *p*-value according to a two-sided hypothesis testing (where the null stands for non-significance). The significance levels are: (*) significance at 10%; (**) significance at 5%; and (***) significance at 1%.

The analysis shows that the first reasons for dissatisfaction toward public LTC services are the long waiting list, long procedure for obtaining the service, and the lack of public transport for reaching the services, while the main reason for dissatisfaction toward private LTC services is the high cost of the services.

In general, satisfaction is significantly associated with the use of private home care services, and dissatisfaction is significantly associated with the non-use of private home care and public outpatient services.

The correlation between the use of LTC services and the satisfaction experienced by users was also analyzed (Table 2). The outcome representing the total dissatisfaction with private LTC service is omitted due to collinearity, suggesting it is highly correlated with other predictors and adds no additional information to the model.

**Table 2:** Logistic function: public and private services use on satisfaction.

Predictors	Estimate	SE	*z*-value	Pr(>|z|)
Private services				
Totally unsatisfied	Omitted due to collinearity			
Almost unsatisfied	17.85	23.90	0.747	0.227
Satisfied	2.689	1.129	2.382	0.017**
Very satisfied	3.778	0.461	8.190	0.000***
Totally satisfied	3.296	0.599	5.150	0.000***
Public services				
Totally unsatisfied	17.022	15.651	1.088	0.138
Almost unsatisfied	17.795	23.033	0.773	0.221
Satisfied	18.509	11.041	0.017	0.987
Very satisfied	5.010	1.0268	4.879	0.000***
Totally satisfied	4.232	1.0393	4.072	0.000***

Note. Here, ‘Coefficients’ refers to the covariates; ‘Estimate’ refers to the estimated best final predictors; ‘SE’ stands for Standard Error; ‘z-value’ denotes the test statistic obtained for each predictor (the ratio between ‘Estimate’ and ‘SE’); and ‘Pr(>|z|)’ refers to the associated *p*-value according to a two-sided hypothesis testing (where the null stands for non-significance). The significance levels are: (*) significance at 10%; (**) significance at 5%; and (***) significance at 1%.

In this context, the TBM procedure was specifically designed to systematically address potential collinearity among predictors and mitigate multicollinearity. In the first step of the TBM procedure, Bayesian model selection utilizing the Bayesian Information Criterion (BIC) was employed to identify and retain only those predictors with significant explanatory power, automatically excluding highly collinear variables that did not provide unique explanatory value. This selection process inherently controls collinearity by emphasizing parsimony and penalizing model complexity. In instances where high correlation persisted among predictors, such as the previously mentioned “total dissatisfaction with private LTC services,” preliminary correlation analyses explicitly identified these predictors, which were subsequently omitted to ensure model robustness and avoid redundancy.

Alternative strategies for addressing collinearity, including variance inflation factor (VIF) analysis, ridge or lasso regression methods, and principal component analysis (PCA), were considered. However, these methods were ultimately not adopted due to the specific nature of the data and research objectives. The Bayesian model selection approach was deemed most appropriate as it systematically balances model fit and complexity, ensuring robustness without compromising interpretability and model parsimony.

The outputs “Totally unsatisfied,” “Almost unsatisfied,” and “Satisfied” have extremely high coefficients, despite the fact that they are not statistically significant (*p*= > 0.99).

High and total satisfaction with public services is highly significant, confirming that higher satisfaction levels strongly contribute to service use.

Being almost unsatisfied with both public and private LTC services and satisfied with public ones has minimal influence on the use of the services.

With a normalized contribution of 97%, satisfaction with private LTC services dominates the other predictors (Table 3), suggesting that satisfaction at this level is the most influential factor in determining the outcome, likely reflecting a critical threshold in service perception.

**Table 3:** Normalized contribution (%): public and private services use on satisfaction.

Variable	Normalized contribution (%)*
Private LTC services	
Satisfied	97
Almost unsatisfied	48.98
Totally unsatisfied	22.62
Very satisfied	0.14
Totally satisfied	0.06
Public LTC services	
Almost unsatisfied	51.58
Very satisfied	0.04
Very satisfied	0.02
Satisfied	0.01

The normalized contribution is a relative measure of the impact of each predictor compared to the one with the highest effect. It provides a scaled value (0 to 100%) to indicate the relative importance of predictors. *Formula: $\mathrm{Normalized}\;\mathrm{Contribution}=\left[\left(\mathrm{Odds}\;\mathrm{Ratio}-1\right)/\left(\max\;\mathrm{Odds}\;\mathrm{Ratio}-1\right)\right]\times 100$ .

Being almost unsatisfied with public and private LTC services, older people and their family caregivers moderately influence the use of the services by (51.58 and 48.98%, respectively).

Dissatisfaction with private LTC services may have some influence (22.62%), but it is less impactful than higher satisfaction levels. This suggests that dissatisfaction in the private care sector may matter and that its influence may diminish compared to moderate or high satisfaction levels.

High and total satisfaction with both private and public services contribute very little to the use of LTC services (all below 0.15%). This might indicate a diminishing return effect, where higher satisfaction levels (e.g., high significance levels in the logistic function) do not significantly alter the tendency of using a service once a certain threshold of satisfaction is reached (e.g., “Satisfied”).

### 3.3 Drivers and barriers to the use of elder LTC services

Concerning the drivers to the use of public healthcare services (Table 4), there is a high statistical significance between the use of the services and the OCR’s age (*p* = 0.000; OR = 1.438), the perceived health status (*p* = 0.000; OR = 1.559), the FC’s age (*p* = 0.009; OR = 1.451), the non-use of private services (because too much expensive), and financial difficulties (*p* = 0.000; OR = 1.614).

**Table 4:** Logistic function: drivers and barriers to the use of healthcare services.

Predictors	Estimate	SE	*z*-value	Pr(>|z|)	OR	Effect (%)
Drivers						
OCR’s age	0.363	0.082	4.445	0.000***	1.438	43.72
OCR’s perceived health	0.444	0.100	4.457	0.000***	1.559	55.81
FC’s age	0.372	0.158	2.354	0.009***	1.451	45.06
FC’s no use of private service (Too much expensive)	0.670	0.302	2.220	0.000***	1.954	95.48
FC’s financial difficulties	0.479	0.062	7.685	0.000***	1.614	61.27
OCR’s dependency (Barthel Index)	0.622	0.296	2.098	0.018**	1.863	86.25
MCW’s Evaluation (too much expensive)	0.652	0.340	1.917	0.027**	1.919	91.93
FC’s no use of public service (no access requirements)	0.610	0.294	2.073	0.019**	1.840	84.06
FC’s use of service	0.493	0.252	1.958	0.050*	1.637	63.77
FC’s importance of proper information (much/very)	0.509	0.353	1.444	0.075*	1.664	66.43
Barriers						
OCR’s wellbeing (WHO-5 Index)	−1.365	0.158	−8.664	0.000***	0.255	74.45
Employment of an MCW	−0.597	0.326	−1.830	0.034**	0.550	81.61
MCW’s country						
Eastern Europe	−0.256	0.092	−2.802	0.003***	0.774	22.62
Extra EU countries	−0.332	0.084	−3.939	0.000***	0.717	28.23
FC- self-employed	−0.896	0.115	−7.821	0.000***	0.408	59.16
Informal support received (family caregivers, friends, and neighbor)	−1.247	0.624	−1.999	0.023**	0.287	71.25
FC’s use of paid care leaves	−0.566	0.297	−1.141	0.028**	0.568	43.24
FC’s gender (female)	−0.591	0.264	−2.234	0.013**	0.554	44.60
Support received by MCW	−0.725	0.460	−1.576	0.057*	0.484	51.57

“Coefficients” refers to the covariates; “Estimate” refers to the estimated best final predictors; “SE” stands for Standard Error; “*z*-value” denotes the test statistic obtained for each predictor (the ratio between “Estimate” and “SE”); “Pr(>|z|)” refers to the associated *p*-value according to a two-sided hypothesis testing (where the null stands for non-significance); Odds Ratio correspond to the exponential of the estimates; and Effect (%) refers to the variation of the odds of y = 1 given a one-unit increase of X. The significance levels are: (*) significance at 10%; (**) significance at 5%; and (***) significance at 1%.

A moderate positive correlation is observed between the use of services and: the OCR’s level of dependency (*p* = 0.018; OR = 1.863); the evaluation of the MCW as too much expensive (*p* = 0.027; OR = 1.919); and the lack of access requirements to the public services (*p* = 0.019; OR = 1.840). Using other services (*p* = 0.050; OR = 1.637) and attributing high importance to receiving proper information (*p* = 0.075; OR = 1.664) are only slightly correlated to the use of services.

The main barriers to the use of services (Table 4) are OCR’s good levels of wellbeing (*p* = 0.000; OR = 0.255), FC’s self-employment (*p* = 0.000; OR = 0.408), and employing an MCW (*p* = 0.003; OR = 0.774).

Receiving informal support from relatives and friends and receiving support from an MCW are moderately correlated to the non-use of services (*p* = 0.023; OR = 0.287 and *p* = 0.034; OR = 0.550, respectively).

Noteworthy, the significance varies according to the MCW’s birth country: care workers from no EU country (*p* = 0.0000) and care workers from Eastern Europe (*p* = 0.0026) but with slight effects on the outcome (22.62 and 28.23%, respectively).

Not benefiting from paid care leaves by FCs, according to law 104/1992, is moderately significantly correlated to a reduced use of services (*p* = 0.028; OR = 0.568) with an effect set to 43.24%.

Table 5 shows that the increased frequency of outpatient services (e.g., general practitioner and nurse) would require the use of additional services (*p* = 0.000), especially help with house cleaning and persons’ care. The difficulty in finding these services (economic and non-economic) demonstrates the positive effect of the covariate (e.g., greater demand for outpatient services). Moreover, the increase in dissatisfaction would lead to a need for more services (*p* = 0.000).

**Table 5:** Logistic function: predictors of use of healthcare services.

Predictors of use of LTC services	Estimate	SE	*z*-value	Pr(>|z|)
Frequency in the use of outpatient services	2.011	0.316	6.362	0.000***
Dissatisfaction with the use of outpatient services	−1.138	0.356	−3.198	0.000***
Dissatisfaction with outpatient services	−1.118	0.652	1.713	0.087*
Frequency in the use of public home care services	−2.346	0.581	4.035	0.000***
Dissatisfaction with public home care services	3.320	0.437	7.596	0.000***
Frequency in the use of private	3.148	1.108	2.841	0.005***
Satisfaction with the use of private services	3.943	0.782	5.041	0.000***
Dissatisfaction with the use of private services	1.512	0.486	3.115	0.000***

Here, “Coefficients” refers to the covariates; “Estimate” refers to the estimated best final predictors; “SE” stands for Standard Error; “*z*-value” denotes the test statistic obtained for each predictor (the ratio between “Estimate” and “SE”); and “Pr(>|z|)” refers to the associated *p*-value according to a two-sided hypothesis testing (where the null stands for non-significance). The significance levels are: (*) significance at 10%; (**) significance at 5%; and (***) significance at 1%.

Moreover, the low frequency in the use of public home care (ADI) increases the need for further services (*p* = 0.000). The low utilization of this is correlated to the non-satisfaction with the quality/quantity of the service (*p* = 0.000). For instance, under “other,” the main responses have been “excessive costs” for private home care. More precisely, according to the few responses received (70 out of 441), the costs incurred are on average 1,121€ per month (The datum is not reported in Table 5). Older people would like more private services, such as day-care centers and transport (*p* = 0.005). The reason for dissatisfaction is more related to the poor quantity/quality of services and to the costs (*p* = 0.000).

## 4 Discussion and conclusion

This is one of the few studies exploring the LTC needs of older people and family caregivers living in a region of Central Italy that involves care dyads and one of the first that identifies the barriers and drivers to the use of healthcare and LTC services at the regional level.

The study confirms that the barriers to the use of LTC and healthcare services by older people and their family caregivers in this region have their origin at micro, meso, and macro societal levels, and that they are often intertwined and reciprocally dependent.

At the macro level, the study shows that the poor offer of public home care and day-care centers, and the unaffordable costs of the private ones are the main barriers to the use of public LTC and healthcare services. This result mirrors the lack of an all-embracing LTC system in Italy, which is able to take inequalities under control (24, 27).

At the micro level, the main barriers are the family caregiver’s age and gender and the older person’s income. The use of LTC services increases with the family caregiver’s age increase, and mirrors a bulk of literature on the prevalence of women in informal caregiving (47), especially in personal care (48). Moreover, the study demonstrates that older care recipients with a lower income tend to make higher use of public healthcare services (e.g., day-hospital and general practitioners) that are for free. Noteworthy, an intensive use of public outpatient services does not correspond to a higher personal satisfaction with the service. In other words, respondents do not use a service because they are happy with it, but they are unhappy with it. This dissatisfaction might be due to the greatest difficulties they reported in accessing services (e.g., bureaucracy, transportation, and waiting lists). These outcomes confirm previous findings focused on the Italian context (38, 39), and also add the lack of public transport for reaching services to the list of already well-known barriers. In fact, outpatient visits and clinical and screening examinations are offered throughout the Marche region without following the principle of proximity to the patient’s home. Thus, it can happen that the first facility available to carry out the examination needed is 50 or more km away, in places that can often only be reached by car. This system of providing medical services puts at a disadvantage the older persons who do not feel safe driving and persons with disabilities who may be dependent on others for transportation. This difficulty is overcome by accessing closer and more expensive private services, but only by citizens who can afford them.

At the meso level, the main barriers to the use of public LTC services are the scarcity of services and the low level of satisfaction with them (24, 27), the lack of proper information on the features of the services, and the long and difficult access procedure also through the local offices (29).

Among the meso-level factors influencing the use of services, we also include the employment of migrant care workers (MCWs), as these represent the nexus between public resources (i.e., IDA) and private households. In contrast to García-Gómez et al. (55), our study underlines that considering the employment of an MCW as too expensive does not prevent families from hiring them. In fact, the families’ need for daily intense care of OCRs and the poor and insufficient provision of public home care services make MCWs an indispensable support that FCs are willing to pay for. Moreover, employing an MCW is still cheaper than resorting to a residential facility (approximately 1,000 vs. circa 2,000 Euros/month).

Noteworthy, the employment of an MCW, associated with the high satisfaction reported toward this service, coincides with a higher use of public LTC services. This outcome can probably be explained by the fact that older people with medium-high dependency levels (i.e., this study’s inclusion criteria) integrate the few existing public LTC services, such as home care (ADI), with the constant and tailored support provided by the MCW paid out-of-pocket because this is considered well-spent money.

The study also sheds light on the small difference in the full satisfaction with this private service, depending on the MCW’s country of origin. In fact, older persons who can count on the support of an MCW from a non-EU country tend to be more satisfied than those who hired an MCW from European countries (especially Eastern ones). This outcome can be explained by the different migration plans of the two typologies of care workers. MCWs from outside Europe live permanently in Italy and rarely reach their country of origin. This may increase their attachment to the job and give family caregivers greater guarantees of continuity in the care job. In contrast, MCWs from Eastern Europe tend to return to their home country more often, thus forcing family caregivers to find substitutes for their periods of absence (16, 49).

### 4.1 Policy considerations: the long way toward an all-embracing LTC system in Italy

In light of the results, some considerations follow on whether and to which extent the current reform of the LTC sector in Italy can address the real needs of older adults and their family caregivers and break down the barriers to the use of healthcare and LTC services.

The results confirm that home care is the most needed service among the surveyed older Italians and that the first obstacle to the use of domiciliary care (ADI) is its unavailability or the low number of hours and intensity of assistance currently granted by the Marche’s regional health system. Unfortunately, the first implementing decree of Law 33/2023, as approved in March 2024, did not indicate how to increase the number of hours of home care provided and its healthcare intensity, nor on the professionals involved. Only the coordination between social and health interventions provided by the current home care services remains mentioned, while decisive aspects such as the duration of the care provided and the different professionals to be involved have been so far neglected. It is therefore urgent that policymakers come back to the reform and consider providing clear guidelines and indicating proper resources for improving this service. In addition, it is worth mentioning that in this first implementing decree, the functions of the SNAA (i.e., the body entitled to unify health, social, and welfare measures at different government levels) were limited to the management of social services only (thus neglecting the health ones and INPS benefits), excluding the local, regional authorities entitled to provide healthcare services and the local ones that are in charge of social services provision.

This study, in line with previous literature (50), shows that the employment of MCWs remains the main strategy for facing the insufficient provision of domiciliary care in the Marche region, mirroring the national trend. Thus, policies strengthening the training of MCWs and integrating them into a system of matching labor supply and demand (51) are recommended to increase their bargaining power, ensuring fair contractual treatment and improving the quality of care provided (17, 18). The Law 33/2023 provides for recognition of social security and tax benefits with a view to their “reorganization,” i.e., tax intervention (e.g., deductions) to relieve families somewhat of the burden of paying for personal assistance. The law also foresees the training of the MCWs and the identification of training standards to address, and from which training plans and regional registers should be developed. Nevertheless, the implementing decrees did not give indication either about the tax intervention for older adults or the training standards for MCWs, postponing these issues to subsequent decrees.

The study also shows that benefitting from the paid leaves established by Law 104/1992 is associated with a lower use of other services by FCs, thus highlighting a strong connection between the macro (the national legislation) and the micro societal level (the individual choices). In fact, according to this regulation, all FCs of older persons who do not reach a level of severe or very severe disability but still need assistance (because they have lost a large part of their autonomy) are excluded from the use of paid care leaves and home care services (mainly the ADI). Paid leaves modulated on different degrees of disability and the possibility of choosing between in-kind services and monetary contribution continue to be essential not to cutoff millions of older people with partial LTC needs and working family caregivers who daily fight for properly reconciling work and care. This aspect, as well as measures to support family caregivers, such as training, psychological support, respite care etc., have been totally neglected by the first implementing decree, which provides only generic indications on support via new regulations, measures to certify professional skills acquired, and of their participation in service planning.

Moreover, concerning residential care, the implementing decree does not contain any substantial indications and refers to a later decree. On the contrary, the inequality in the availability of beds in residential facilities and the quality of the provided assistance across regions (52) would require a clear intervention by the State in accordance with the regional healthcare authorities that participate to covering healthcare costs.

Moreover, the IDA was not reformed at all, but a new universal cash benefit was added, which will be tested in the 2 years 2025/2026 and consists of increasing the IDA by approximately 250%. This means that a small, highly selected sample of 25,000 over 80-year-old people, already entitled to receive the IDA according to the past legislation, will receive 1.380€ instead of the 530€ they currently receive until the allocated funds are exhausted. Furthermore, the lack of clearness about available services and procedures represents a strong barrier to access to public health services. This result calls for opening and/or strengthening Single Points of Access (Punti Unici di Accesso) and providing individualized care plans (Piani Assistenziali Individualizzati) for each user based on the multidimensional assessment, as foreseen by Law 33/2023. Again, the first implementing decree, in fact, reaffirms the basic principle but does not indicate how to implement it, i.e., in terms of the composition of the assessment unit, how it is to operate, the national tool for assessing LTC, and disability conditions.

Concerning the correlation between the use of public and private LTC services and the satisfaction with their use, high levels of satisfaction seem to be a driver to the use of LTC services by older people and family caregivers, to a higher extent than the satisfaction with private services, thus highlighting a specific area for targeted improvement in public services.

Moreover, the provision of outpatient services, such as specialist visits and screening examinations, has to be re-designed to make services more accessible, closer to the patients’ home, and reachable with public transport. To this purpose, agreements with transport companies and voluntary associations could be signed to maintain the costs affordable and overcome inequalities for (older) people with severe physical and/or cognitive limitations.

Noteworthy, for the dependent older people and their family caregivers in Italy, it is enough to have a minimum level of satisfaction with public LTC services to continue using them. This indicates a downward adjustment of their care expectations, which could also hide a kind of resignation. A similar attitude is found for using private LTC services, i.e., mainly the employment of MCW. In fact, dissatisfaction weighs less heavily than satisfaction in using private services such as MCW. Thus, although an older person is dissatisfied, s/he will continue to employ MCW, probably because there is no alternative that provides greater satisfaction for the same cost and quality of care provided. This outcome also calls for an effort by the regional healthcare system to increase the quality level of public LTC services to meet the care expectations of their citizens. To improve the chances that the services meet the users’ preferences, the regional healthcare systems manager should foresee co-design sessions with representatives of older people with LTC needs and their caregivers.

Thus, to date, not all the needs of the older people have been met by the reform, and there are still factors hindering the use of public LTC services, such as the scarcity of in-kind measures provision, clear information, services reachable by citizens with limited mobility solutions, especially older people, support services for family caregivers, the regulation of MCWs employment and their life-long training ensuring the quality of the care provided, and a tested-means monetary contribution based on different level of disability. It is crucial to underline such barriers to design evidence-based policies and measures both at the national and regional levels that can be integrated in future decrees, to implement the point of the reform that have not been met yet. Under current circumstances, however, the way toward a universal and comprehensive LTC system that can interpret at the national level the needs of the older population and family caregivers raised at the local level seems to be still rather long.

### 4.2 Study limitations and suggestions for future research

This study is not without limitations. The main one lies in the convenience/purposive and small-size sampling that does not allow data generalization to the overall older Italian population. However, it is precisely the specificity of this sample that can provide an in-depth snapshot of what is happening in this region and contribute to an understanding of the differences and disparities that can exist within the same country.

Moreover, the questionnaire did not include questions on respondents’ cultural patterns, such as, filial obligations, stigma, and traditional representations of care that might have influenced the access and use of healthcare and LTC services.

Furthermore, the use of open-ended questions would have allowed the collection of respondents’ opinions on the quality of services, the difficulties they face, and their suggestions for improvement.

Finally, the questionnaire did not explore the respondents’ expenditure for private healthcare and LTC services, thus preventing any analysis of cost barriers to private LTC services.

From a research perspective, other region-based studies are welcome to compare different regional health systems’ weaknesses and strengths. Moreover, longitudinal and cohort studies are recommended to understand the effects of the LTC reform on the use of services: the same questionnaire should be administered to the older population with the same characteristics as the one involved in this study, between 5 and 10 years. Moreover, regional-based studies that analyze older care recipients’ perspectives using a qualitative method can help better understand the difficulties they encounter in everyday care.

The results of this study confirm the initial hypothesis that the use of LTC services is shaped by a combination of factors operating at macro, meso, and micro levels. However, findings also suggest alternative interpretations that deserve further attention. For instance, the employment of migrant care workers, often seen as a substitute for public provision, appears here as a complementary strategy to compensate for gaps in formal care. Furthermore, the influence of satisfaction on service use highlights a behavioral dimension that may reflect adjusted expectations or resignation, particularly among older adults. These insights call for future research and policy efforts to reconsider how user experience and informal care arrangements interact with formal service delivery.

## Data Availability

The data analyzed in this study is subject to the following licenses/restrictions: The data presented in this study are available upon request from the corresponding author. The data are not publicly available because they are still the object of ongoing analyses. Requests to access these datasets should be directed to antonio.pacifico@unimc.it.
