# Supplementary material for: Reforming Italy’s long-term care system: the role of barriers to and drivers of the use of services at the local level
Source: Front Public Health. 2025 Apr 28;13:1575330. doi: 10.3389/fpubh.2025.1575330 (PMC12066302; doi:10.3389/fpubh.2025.1575330)
Supplement: Supplementary file 1 [file Table_1.docx]

Supplementary Material

Table 1: *Family caregivers and older care recipients’ socio-demographic characteristics*

| Family carers (Migrant Care Workers) | N(%) |
| --- | --- |
| Age (Mean ±sd) | 65.74±13.37 |
| Gender |  |
| Males | 126 (28.57) |
| Females | 315 (71.43) |
| Citizenship |  |
| Italian | 388 (96.76) |
| Albanian | 1(0.25) |
| Indian | 2 (0.50) |
| Moroccan | 1(0.25) |
| Moldavian | 2(0.50) |
| Polish | 1(0.25) |
| Romanian | 4(1.00) |
| Russian | 2(0.50) |
| Marital Status | |
| Married/Cohabitant | 277(69.25) |
| Widowed | 22(5.50) |
| Divorced/Separated | 38(9.50) |
| Unmarried | 64(16.00) |
| Educational Level | |
| None | 6(1.50) |
| Primary school | 60(14.96) |
| Secondary school | 110(27.43) |
| High school | 176(43.89) |
| Post-secondary school (non-tertiary) | 2(0.50) |
| Higher education (tertiary) | 4(1.00) |
| Bachelor degree | 7(1.75) |
| Master degree | 34(8.48) |
| Ph.D. | 2(0.50) |
| Relationship with the older care recipient |  |
| Spouse | 83(20.75) |
| Son/Daughter | 242(60.50) |
| Brother/Sister | 13(3.25) |
| Daughter/Son in-law | 26(6.50) |
| Other (including MCWs) | 36(9.00) |
| Living condition in respect of the OCR |  |
| In the same apartment | 229(56.97) |
| Different apartments but same building o | 50(12.44) |
| Within walking distance | 66(16.42) |
| 10 minutes by car, bus or train | 44(10.95) |
| 30 minutes by car, bus or train | 11(2.74) |
| No more than 1 hour by car, bus or train | 1(0.25) |
| More than 1 hour by car, bus or train | 1(0.25) |
| Period (in months) assisting the old person | 85.83±6.31 |
| Hours per week (on average) assisting the older person | 62.06±77.78 |
| Working caregivers | 125(28.34) |
| Older care recipients |  |
| Age (Mean ±sd) | 85.83±6.31 |
| Gender |  |
| Males | 126(28.57) |
| Females | 315(71.43) |
| Citizenship |  |
| Italian | 439(99.55) |
| Moroccan | 1(0.23) |
| Albanian | 1(0.23) |
| Marital Status |  |
| Married/Cohabitant | 152(34.47) |
| Widowed | 260(58.96) |
| Divorced/Separated | 3(0.68) |
| Unmarried | 26(5.90) |
| Educational Level |  |
| None | 62(14.06) |
| Primary school | 273(61.90) |
| Secondary school | 60(13.61) |
| High school | 29(6.58) |
| Post-secondary school (non-tertiary) | 0(0.00) |
| Higher education (tertiary) | 1(0.23) |
| Bachelor degree | 4(0.91) |
| Master degree | 8(1.81) |
| Living condition |  |
| Alone | 66(14.97) |
| With spouse/partner | 155(35.15) |
| With daughter/son (in-law) | 163(36.96) |
| With MCWs | 121(27.44) |
| Other (daughter in-law; brother/sister in-law) | 38(8.62) |
| Health Condition |  |
| Excellent | 1(0.23) |
| Very good | 8(1.81) |
| Good | 32(7.26) |
| Fair (Reasonable) | 174(39.46) |
| Bad | 93(21.09) |
| Dependency rate (Barhel Index) |  |
| 21-60 | 26.56% |
| 61-90 | 17.58% |
| >=91 | 55.86% |
| Employment of a MCW | 199(45.12) |

*Note: Frequencies and percentage may not correspond to the overall number and percentage of the respondents in case of missing values and when more than one answer was possible.*
